# Genetic polymorphisms of Ca^2+^ transport proteins and molecular chaperones in mitochondria-associated endoplasmic reticulum membrane and non-alcoholic fatty liver disease

**DOI:** 10.3389/fendo.2022.1056283

**Published:** 2023-01-04

**Authors:** Zongzhe Tang, Yajie Ding, Ru Zhang, Mengting Zhang, Qing Guan, Liuxin Zhang, Hongliang Wang, Yue Chen, Rong Jiang, Wei Zhang, Jie Wang

**Affiliations:** ^1^ Department of Fundamental and Community Nursing, School of Nursing, Nanjing Medical University, Nanjing, China; ^2^ Department of Neurosurgery, The Affiliated Brain Hospital of Nanjing Medical University, Nanjing, China; ^3^ Department of General Practice, Ninghai Road Community Health Service Center, Nanjing, China; ^4^ Department of Epidemiology, Shanghai Cancer Institute, Shanghai, China

**Keywords:** non-alcoholic fatty liver disease (NAFLD), heat shock 70-kDa protein 5 (HSPA5), inositol 1,4,5-trisphosphate receptor type 2 (ITPR2), genetic polymorphism, calcium homeostasis

## Abstract

**Background:**

Non-alcoholic fatty liver disease (NAFLD) is recognized to be closely associated with endoplasmic reticulum stress and mitochondrial dysfunction, while previous studies have emphasized the important role of calcium homeostasis from the mitochondria-associated endoplasmic reticulum membrane (MAM) in the endoplasmic reticulum and mitochondria. This article will assess the association between genetic polymorphisms of Ca^2+^ transport proteins and molecular chaperones in MAM and NAFLD risk.

**Methods:**

A case-control study was conducted in a community of Nanjing, China during April to December 2020. 2701 subjects were enrolled and genotyped for 6 genetic variants in *HSPA5* and *ITPR2* genes. Logistic regression analysis was used to assess impact of these variants on NAFLD risk.

**Results:**

After adjusting for age, gender, total cholesterol and glucose, we identified that *HSPA5* rs12009 variant genotypes (recessive model: OR= 0.801, 95% CI= 0.652-0.986, *P*= 0.036), rs430397 variant genotypes (recessive model: OR= 0.546, 95% CI= 0.314-0.950, *P*= 0.032), and *ITPR2* rs11048570 variant genotypes (recessive model: OR= 0.673, 95% CI= 0.453-0.999, *P*= 0.049) were associated with a reduced risk of NAFLD. Multivariate stepwise regression analysis indicated that gender, glucose, body mass index, triglycerides and favorable alleles were independent influencers of NAFLD (all *P*< 0.05). The area under the receiver operating characteristic curve was 0.764 (95% CI= 0.745-0.783, *P*< 0.001).

**Conclusion:**

The variant genotypes of Ca^2+^ transport-associated genes *HSPA5* (rs12009 and rs430397) and *ITPR2* (rs11048570) might contribute to the reduction of the NAFLD risk in Chinese Han population, which can provide new insight into NAFLD pathogenesis.

## 1 Introduction

Non-alcoholic fatty liver disease (NAFLD) has become the leading chronic liver disease, burdening around 24% of the global population (1). Based on recent studies, the prevalence of NAFLD in China is approximately 29.1%-29.8%, similar to the overall level in Asia (29.6%) and gradually approaching that in many Western countries (1–4). NAFLD is an umbrella term that often manifests as liver steatosis in the initial stage, which can spread to non-alcoholic steatohepatitis (NASH), the latter may progress to advanced liver fibrosis, cirrhosis, or hepatocellular carcinoma (HCC) (5). Over the last decades, the growing evidences have shown that NAFLD is a multisystem disease, affecting extra-hepatic organs and regulatory pathways (6), such as type 2 diabetes mellitus (T2DM), cardiovascular disease (CVD) and chronic kidney disease (CKD). Overall, NAFLD contributes to an increasing clinical burden worldwide and may pose a serious threat to people’s health.

Although steady progress has been made in clarifying NAFLD pathogenesis, targeting therapeutics, and facilitating drug development, hard challenges remain. Because of the complex physiological mechanisms and apparent heterogeneity of disease phenotypes, early prevention and healthy lifestyle remain crucial to many patients with NAFLD (7). Besides, there is no standard NAFLD-specific therapy, although some drugs are in advanced stages of development (8). Consequently, it is essential to further investigate the pathogenesis of NAFLD for its early prevention and drug development. the currently well-established “multiple-hit” theory suggests that multiple injuries interacting with genetically susceptible subjects could induce NAFLD, including insulin resistance (IR), endoplasmic reticulum stress (ERS), mitochondrial dysfunction, gut microbiota, nutritional factors, hormones secreted by adipose tissue, as well as genetic and epigenetic factors (9). Meanwhile, the “multiple-hit” theory considers that persistent ERS and mitochondrial dysfunction are important triggers for the development of NAFLD, while IR is central to the pathogenesis of NAFLD (9). In particular, the endoplasmic reticulum and mitochondria are closely related to calcium homeostasis, and the exchange of substances between them and calcium transport has been demonstrated to be associated with NAFLD (10).

Calcium (Ca^2+^), as the most plentiful mineral in the body, is involved in all important cellular functions at the cellular level, such as cell growth, differentiation, metabolism, motility and gene expression (11). In the context of metabolic regulation, Ca^2+^ is required for the insulin and glucagon secretion by the pancreas. Conversely, these hormones also control metabolic reactions in target tissues, such as glycogen breakdown, lipid biosynthesis, gluconeogenesis and ATP production, in a Ca^2+^-dependent manner. Accordingly, the abnormal Ca^2+^ signaling pathway is thought to be one of the contributing factors to the occurrence of hepatic steatosis (12). Particularly, abnormal Ca^2+^ transport in the mitochondria-associated endoplasmic reticulum membranes (MAM) region is closely associated with IR, ERS, and mitochondrial dysfunction (13). Since the MAM region is widely involved in cellular activities such as lipid synthesis and transportation, regulation of mitochondrial dynamics, inflammatory vesicle formation, ERS, autophagy and apoptosis (14), which in turn may have important effects on the occurrence and progression of NAFLD. Moreover, recent studies have implicated that Ca^2+^ signaling in the MAM region can influence hepatic gluconeogenesis, adipogenesis, inflammation, and other biological processes affecting overall metabolism (15), and has been widely explored in the pathogenesis of metabolic diseases such as obesity and T2DM (16). Therefore, we hypothesize that Ca^2+^ signaling in the MAM region may play an important role in NAFLD pathogenesis.

Growing evidence indicates that numerous proteins and genes involved in Ca^2+^ signal transduction are present in the MAM region (17), where Ca^2+^ transport proteins and molecular chaperones can interact to form a Ca^2+^ transport protein cluster (18). The more common of those are the inositol triphosphate receptor-2 (IP3Rs) on the endoplasmic/sarcoplasmic reticulum and the voltage-dependent anion channel (VDAC) on the outer mitochondrial membrane, containing the encoded protein IP3R2 (gene *ITPR2*) and the Ca^2+^ transport protein VDAC1 (gene *VDAC1*) (17). It has been found that IP3R2 in the MAM region interacts with VDAC1 and enhances the formation of the IP3R2-VDAC1-MICU1 complex, which in turn triggers mitochondrial Ca^2+^ overload and early hepatic IR (19). Moreover, some other studies have revealed the role of contacts and facilitated exchanges between the ER and the mitochondria through the inositol 1,4,5-trisphosphate receptor, type 2 (*ITPR2*) in regulating senescence and aging (20). Meanwhile, the MAM region is enriched with molecular chaperone proteins present on the endoplasmic reticulum or mitochondria involved in the regulation of Ca^2+^ transport proteins, such as glucose-regulated protein 75 (GRP75, gene *HSPA9*) and 78-kDa glucose-regulated protein (GRP78, gene *HSPA5*) (21). The chaperone glucose-regulated protein, 78/immunoglobulin binding protein (GRP78/Bip), protects cells from cytotoxicity induced by DNA damage or ERS (22). Indeed, studies indicated that GRP78 may maintain mitochondrial permeability thereby protecting cells from ERS-induced apoptosis (23). Moreover, GRP78 acts as a receptor for a variety of ligands including VDAC, and attenuate ERS (24). In view of the above, polymorphisms of the genes involved in Ca^2+^ transport may affect the onset of NAFLD by regulating Ca^2+^ homeostasis.

This study aimed to investigate the correlation between the genetic polymorphisms of Ca^2+^ transporter gene (*ITPR2*, *VDAC1*) and its related chaperones (*HSPA5*, *HSPA9*, *SIGMAR1*, *CANX*, *PPID*) and NAFLD in Chinese population, to provide new ideas for further exploring the genetic susceptibility mechanism of NAFLD. And this study screened new biomarkers for NAFLD risk prediction and further provided theoretical basis for screening and early intervention of NAFLD high-risk populations.

## 2 Methods

### 2.1 Study participants and design

The sample included NAFLD patients and healthy controls recruited from a certain community in Nanjing during April to December 2020, and the research team recorded and linked with the medical information management center and the chronic disease management department of the district, and screened out the eligible target population according to the diagnosis and admission criteria. All participants gave informed consent.

The inclusion criteria were as follows: (1) Han Chinese; (2) age ≥18 years old with informed consent; (3) those who are capable of giving fasting blood samples; (4) those who are able to communicate and complete questionnaires. Exclusion criteria were: (1) those with infection, acute and chronic autoimmune diseases, malignant tumors; (2) those with a history of viral hepatitis, primary liver cancer, alcohol-related liver disease and other liver diseases; excessive drinking (male ≥30g/d, female ≥20g/d); (3) those who were expected to undergo liver transplantation within one year, or those who had complications such as variceal bleeding or ascites in the advanced stage of liver disease; (4) those with type 2 diabetes, obesity and metabolic syndrome; (5) those who had used drugs or present exacerbated steatohepatitis (e.g., glucocorticoids); (6) patients with a history of psychiatric disorders.

NAFLD cases were diagnosed according to the Guideline of prevention and treatment for non-alcoholic fatty liver disease: a 2018 update (25). The following criteria were specifically required: (1) no history of alcohol consumption or average alcohol consumption < 30g per day for men and < 20g per day for women; (2) exclusion of specific diseases that can result in fatty liver, including viral hepatitis, total parenteral nutrition, hepatomegaly and drug-related liver disease; (3) histological changes on liver biopsy were consistent with the pathological diagnostic criteria for fatty liver disease. Given the difficulty in acquiring a hepatic histological diagnosis, in addition to the first two points, NAFLD could also be diagnosed if any one or two of the following features were present: (i) the hepatic imaging manifestations meet the imaging diagnostic criteria for diffuse fatty liver and there is no other explanation; (ii) patients with metabolic syndrome-related components, such as visceral obesity, hyperglycemia, blood lipid disorder, and hypertension, presenting with unexplained serum alanine aminotransferase (ALT) and/or serum aspartate aminotransferase (AST) and γ-glutamyl transpeptidase (GGT) for more than six months. The controls were collected from the same community during the study period and randomly assigned to the control group by the frequency matching method. Namely, the control group was proportionally matched to the case group based on the same distribution characteristics of age ( ± 3 years) and sex to ensure that the matching factors were distributed in the same proportion in both groups.

Based on the review of literature, we estimated the frequency of gene variant in the general population was 10%-20%, odds ratio (OR) was 1.5, two-tailed test a was 0.05, and test power (1-b) was 80%. Finally, it was estimated by the NCSS-PASS 11 software (Dawson version; Kaysville, Ut) that the minimum sample size was 534. This study had a sample size large enough to guarantee the production of reliable results.

The study was conducted in accordance with the World Medical Association Declaration of Helsinki on Ethical Principles for Medical Research Involving Human Subjects and was approved by the Institutional Ethics Review Committee of Nanjing Medical University (Nanjing, China). Written informed consent was obtained prior to blood testing and genetic analysis.

### 2.2 Clinical information and blood sample collection

The demographic characteristics and clinical information of all participants were obtained from self-designed scales and electronic medical records. All participants underwent abdominal ultrasound and blood biochemical tests. 5-ml of EDTA-anticoagulated venous blood was collected from the study subjects under fasting conditions in the morning. Centrifuged within 2 hours according to standard methods (4,000 g/min, 10 min), and subsequently serum, leukocytes and erythrocytes were separated into 1.5-ml centrifuge tubes (two copies of each were kept) and frozen at -80°C. The serum was used for further blood biochemical and liver function index tests, while the whole blood was used to extract genomic DNA by magnetic bead method and stored at -20°C for later genotyping tests.

### 2.3 SNP selection and genotyping

Target single nucleotide polymorphisms (SNPs) screening was performed in the following steps (1): Aiming to investigate expression patterns of Ca^2+^ transporter protein genes from MAM that may be involved in human liver tissues, database queries and interrogations were performed on the Human Protein Atlas database (https://www.proteinatlas.org/), which reports both protein production and gene expression in different tissues and organs; we also searched the Genotype-Tissue Expression Project (GTEx) (https://gtexportal.org/home/) and the Function Annotation of The Mammalian Genome 5 (FANTOM5) (http://fantom.gsc.riken.jp/5/), which record the results of gene expression and transcription levels. Based on previous literature data about the MAM region, we searched for proteins involved in Ca^2+^ transport. Some of the search terms are as follows: IP3R1, 2, 3; VDAC1, 2; GRP75; GRP79; CYPD; SIG1R; ERP44; Calnexin; NLRP3, etc. 7 genes (*ITPR2*, *VDAC1*, *HSPA5*, *HSPA9*, *SIGMAR1*, *CANX*, *PPID*) were finally screened according to their gene expression and transcription levels in liver tissues (**Supplementary Table 1**). Keeping track of the literature reports to improve the selection of relevant genes is needed (2). Genotype information of Chinese Han population (CHB) for the above 7 genes was downloaded from the Thousand Genome Project database (http://www.1000genomes.org/) (3). Importing information into Haploview 4.2 (Broad Institute, Cambridge, MA, USA), setting Hardy-Weinberg (H-W) *p*-value cutoff= 0.05, the correlation coefficient *r* ^2^≥ 0.8, the minor allele frequency (MAF)> 0.05 to obtain the corresponding tagSNPs (4). Searching the NCBI dbSNP database (https://www.ncbi.nlm.nih.gov/snp), the RegulomeDB database (http://www.regulomedb.org/), and the SNP Function Prediction database (https://snpinfo.niehs.nih.gov/snpinfo/snpfunc.html) and other websites in order to screen out taqSNPs with potential functional significance. Finally, 6 tagSNPs (*HSPA5*-rs1140763, rs12009, rs430397 and *ITPR2*-rs10771283, rs11048570, rs2230372) were selected for genotyping (**Supplementary Table 2**). The genotyping assay was performed by TaqMan-based real-time quantitative polymerase chain reaction technique (Applied Biosystems, USA) and on the Light Cycler 480 II Real-Time PCR System (Roche, Switzerland).

The following measures were applied to control the data quality; (1) genotyping was blinded so that all technicians were ignorant of the participants’ clinical data; (2) replicate experiments were performed in a 10% random sample with a 100% replication rate. All SNPs were genotyped with a success rate above 95%. All trials followed the manufacturer’s instructions.

### 2.4 Statistical analysis

Statistical analyses were performed with SPSS 26.0 (SPSS Inc., Chicago, IL, USA), Stata 16.0 (StataCorp LP, College Station, TX, USA) and genetics software (Haploview 4.2, HAPSTATA 3.0, etc.). Distributions of demographic and clinical characteristics among case and control groups were compared by the Chi-square (*χ* ^2^) test for categorical data, Student’s *t*-test for measurement data, or one-way analysis of variance (ANOVA) when appropriate. Logistic regression analysis was used to calculate odds ratio (OR) and 95% confidence interval (95% CI) to assess the associations between genotypes and risk of NAFLD, adjusting for gender, age, total cholesterol (TC), and glucose (GLU). Each SNP was analyzed using codominant model (mutant homozygous type *vs.* wild homozygous type and heterozygous type *vs.* wild homozygous type, respectively), dominant model (mutant homozygous type+ heterozygous type *vs.* wild homozygous type), recessive model (mutant homozygous type *vs.* heterozygous type+ wild homozygous type), and additive models (mutant homozygous type *vs.* heterozygous type *vs.* wild homozygous type). False discovery rate (FDR) corrections were used for multiple comparisons, considering the *P*_FDR_≤ 0.25 as modest confidence that the correlation represented a positive result (26). The Cochran-Armitage trend test was used to analyze the combined effect of independent positive SNPs. Stratified analysis of positive SNPs was conducted, and heterogeneity between subgroups was calculated by Q-test. Multivariate stepwise logistic regression analysis was performed to determine independent predictors of NAFLD. A receiver-operating characteristic curve (ROC) represented the risk prediction model for NAFLD, with its predictive power expressed as area under the receiver operating characteristic curve (AUROC). The Hardy Weinberg equilibrium (HWE) was tested using a goodness of-fit *χ* ^2^-test among case and control subjects. Haploview software was used to estimate Linkage disequilibrium (LD) parameters (i.e., *D* and *r* ^2^), and PHASE 2.1 software to analyses haplotype frequencies. All statistical analyses were two-sided with a significance level of *P*< 0.05.

## 3 Results

### 3.1 Basic characteristics of study subjects

A total of 2701 subjects were enrolled in this study. The distribution of demographic characteristics and clinical biochemical indices of the subjects are shown in **Table 1**. Although we chose to match the characteristics by gender and age (tolerance error of 3 years) and to select the control group in a ratio of approximately 2:1 with cases, the difference in gender was too large and a substantial sample would be lost if matched completely. We therefore ultimately chose to maximize gender matching on the age-matched results and included gender as confounder in the subsequent data analysis to minimize the effect of confounders on the results. There were no statistically significant differences between the two groups regarding mean age, dietary patterns and dietary preferences (all *P*> 0.05). However, there were statistically significant differences in gender, TC, GLU, triglyceride (TG) and body mass index (BMI) (all *P*< 0.05).

**Table 1:** Comparison of demographic and clinical data between the NAFLD and control groups.

Variables	NAFLD group (n= 815) No. (%)	Control group (n= 1886) No. (%)	*P*
Age (years)	43.40± 9.188	42.74± 7.998	0.078^*^
Gender			< 0.001^**^
Male	731 (89.7)	1226 (65.0)	
Female	84 (10.3)	660 (35.0)	
TC (mmol/L)			0.003^**^
< 5.72	681 (83.6)	1656 (87.8)	
≥ 5.72	134 (16.4)	230 (12.2)	
GLU (mmol/L)			< 0.001^**^
< 5.60	468 (57.4)	1319 (69.9)	
≥ 5.60	347 (42.6)	567 (30.1)	
TG (mmol/L)			< 0.001^**^
< 1.70	434 (79.3)	1495 (79.3)	
≥ 1.70	381 (46.7)	391 (20.7)	
BMI (kg/m^2^)			< 0.001^**^
< 24	222 (28.4)	1213 (67.2)	
≥ 24	561 (71.6)	592 (32.8)	
Dietary patterns			0.317^**^
0	11 (1.6)	14 (0.9)	
1	684 (97.7)	1593 (98.5)	
2	5 (0.7)	11 (0.7)	
Dietary preferences			0.857^**^
0	643 (98.0)	1472 (98.1)	
1	13 (2.0)	28 (1.9)	

NAFLD, non-alcoholic fatty liver disease; TC, total cholesterol; GLU, glucose; TG, triglyceride; BMI, body mass index.

Dietary patterns: 0 indicates meat-based diet, 1 indicates balanced meat and vegetarian diet, 2 indicates vegetarian diet; Dietary preferences: 0 indicates no preference, 1 indicates sugar, salt or oil addiction. The variables TC, GLU, and TG in this table were classified as normal versus abnormal data according to the relevant guidelines. The classification of BMI was based on the level of the Chinese population, and those over 24 kg/m^2^ were considered overweight.

^*^ Student’s t-test.

^**^ Chi-square test. Bold type indicates statistically significant results.

### 3.2 Associations of *HSPA5* and *ITPR2* SNPs with NAFLD risk

Three genetic models (dominant, recessive, and additive models) were applied to elucidate the association between the selected SNPs and susceptibility to NAFLD. The genotype distribution and logistic regression analysis results of the 6 SNPs between the two study groups were shown in **Table 2**. Logistic regression results adjusted for age, sex, TC, and GLU showed that three SNPs (*HSPA5*-rs12009, *HSPA5*-rs430397, and *ITPR2*-rs11048570) were associated with susceptibility to NAFLD (all *P*< 0.05). Compared with subjects carrying the rs12009-GG+GA genotype, those carrying the rs12009-AA genotype had a significantly decreased risk of developing NAFLD (recessive model: adjusted OR= 0.801, 95% CI= 0.652-0.986, *P*= 0.036). Individuals carrying the wild pure genotype rs430397-TT had a significantly reduced risk of NAFLD than those carrying the rs430397-CC genotype (adjusted OR= 0.541, 95% CI= 0.310-0.945, *P*= 0.031). Meanwhile, individuals carrying the rs430397-CC+CT genotype had a significantly lower risk of NAFLD compared to those carrying the rs430397-TT genotype (recessive model: adjusted OR= 0.546, 95% CI= 0.314-0.950, *P*= 0.032). We also found that participants with the rs11048570-AA genotype had a reduced susceptibility to NAFLD compared with the rs11048570-GG genotype (adjusted OR= 0.664, 95% CI= 0.444-0.993, *P*= 0.046). In addition, individuals carrying the rs11048570-AA genotype appear to be less vulnerable to NAFLD compared with the rs11048570-GG+GA genotype (recessive model: adjusted OR= 0.673, 95% CI= 0.453-0.999, *P*= 0.049). FDR correcting for multiple comparisons outcomes were statistically significant (all *P*_FDR_≤ 0.25, **Supplementary Table 3**). However, no significant association was discovered between *HSPA5*-rs1140763, *ITPR2*-rs10771283 and *ITPR2*-rs2230372 variants (all *P*> 0.05).

**Table 2:** Relationship between target gene SNPs and NAFLD.

SNPs	NAFLD group No. (%)	Control group No. (%)	OR (95% CI)	P^*^
*HSPA5*-rs1140763				
GG	247 (31.4)	596 (32.7)	1.000 (ref.)	
GA	394 (50.1)	917 (50.2)	1.088 (0.892-1.327)	0.406
AA	145 (18.4)	312 (17.1)	1.183 (0.914-1.532)	0.202
Dominant model			1.112 (0.921-1.342)	0.269
Recessive model			1.124 (0.895-1.412)	0.314
Additive model			1.088(0.958-1.235)	0.193
*HSPA5*-rs12009				
GG	220 (27.1)	505 (26.8)	1.000 (ref.)	
GA	415 (51.2)	915 (48.6)	1.008 (0.820-1.238)	0.940
AA	176 (21.7)	461 (24.5)	0.806 (0.630-1.031)	0.086
Dominant model			0.939 (0.773-1.140)	0.525
Recessive model			**0.801 (0.652-0.986)**	**0.036**
Additive model			0.902 (0.799-1.019)	0.098
*HSPA5*-rs430397				
CC	531 (65.7)	1216 (64.6)	1.000 (ref.)	
CT	260 (32.2)	599 (31.8)	0.974 (0.809-1.174)	0.784
TT	17 (2.1)	66 (3.5)	**0.541 (0.310-0.945)**	**0.031**
Dominant model			0.928 (0.774-1.113)	0.423
Recessive model			**0.546 (0.314-0.950)**	**0.032**
Additive model			0.893 (0.761-1.047)	0.163
*ITPR2*-rs10771283				
GG	275 (34.2)	617 (33.0)	1.000 (ref.)	
GA	397 (49.4)	951 (50.9)	0.932 (0.768-1.129)	0.470
AA	132 (16.4)	299 (16.0)	1.004 (0.773-1.304)	0.978
Dominant model			0.949 (0.790-1.139)	0.571
Recessive model			1.047 (0.827-1.325)	0.703
Additive model			0.988 (0.870-1.122)	0.851
*ITPR2*-rs11048570				
GG	461 (57.8)	1060 (56.9)	1.000 (ref.)	
GA	301 (37.7)	680 (36.5)	0.969 (0.807-1.163)	0.733
AA	36 (4.5)	123 (6.6)	**0.664 (0.444-0.993)**	**0.046**
Dominant model			0.923 (0.774-1.101)	0.373
Recessive model			**0.673 (0.453-0.999)**	**0.049**
Additive model			0.895 (0.775-1.035)	0.134
*ITPR2*-rs2230372				
GG	319 (39.8)	772 (41.8)	1.000 (ref.)	
GA	365 (45.5)	793 (43.0)	1.108 (0.917-1.338)	0.287
AA	118 (14.7)	281 (15.2)	0.994 (0.764-1.294)	0.967
Dominant model			1.078 (0.903-1.287)	0.406
Recessive model			0.943 (0.739-1.203)	0.634
Additive model			1.021 (0.903-1.156)	0.736

SNPs, single nucleotide polymorphisms; OR, odds ratio; CI, confidence interval.

A pair of alleles such as G/A, if A is a less-frequent allele, then dominant model (AA+ GA vs. GG), recessive model (AA vs. GA+ GG) and additive model (GG vs. GA vs. AA). Bold type indicates statistically significant results.

^*^ Logistic regression model, adjusted for gender, age, TC and GLU.

### 3.3 Combined effect and stratified analysis

As shown in **Table 3**, the combined effect of rs12009-A, rs430397-T, and rs11048570-A on NAFLD was assessed by counting the allele numbers of these SNPs. The results showed that the 3 alleles were favorable alleles, and participants with “4-6” favorable alleles had a significantly lower risk of NAFLD compared to study participants with “0” protective alleles, adjusted OR= 0.575 (95% CI: 0.390-0.848, *P*= 0.005).

**Table 3:** Combined effects of rs12009-A, rs430397-T, and rs11048570-A on NAFLD risk.

Variables^†^	NAFLD group No. (%)	Control group No. (%)	OR (95% CI)	P^*^
0	123 (15.1)	304 (16.1)	1.000 (ref.)	
1-3	640 (78.5)	1392 (73.8)	1.057 (0.831-1.346)	0.650
4-6	52 (6.4)	190 (10.1)	**0.575 (0.390-0.848)**	**0.005**
Trend				P^**^
0	123 (15.1)	304 (16.1)	1.000 (ref.)	
1-6	692 (84.9)	1582 (83.9)	0.997 (0.785-1.267)	0.982

NAFLD, non-alcoholic fatty liver disease; OR, odds ratio; CI, confidence interval.

Bold type indicates statistically significant results.

^†^ The number of favorable alleles: rs12009-A, rs430397-T, and rs11048570-A.

^*^ Logistic regression analysis after adjustment for gender, age, TC, and GLU.

^**^ Cochran-Armitage trend test.

Subsequently, stratified analyses were conducted to eliminate potential biases regarding sex, age, TC, and GLU as a way to further explore the combined effects of rs12009-A, rs430397-T, and rs11048570-A. Considering the reasonableness of results, age stratification was based on the mean and median age of the sample population; the remaining factors were stratified according to data in the “Recommendations for the prevention and treatment of dyslipidemia” “Practice Guidelines (2020) for Integrated Management of Cardiovascular Disease in Primary Hospitals” and “Guideline of prevention and treatment for nonalcoholic fatty liver disease: a 2018 update” (25, 27, 28). After analysis, a significant association between the favorable alleles and the risk of NAFLD could be found in the male subgroup (adjusted OR= 0.786, 95% CI= 0.649-0.952, *P*= 0.014) and in the age> 42 years subgroup (adjusted OR= 0.765, 95% CI= 0.597-0.980, *P*= 0.034), which were showed in **Table 4**. Besides, heterogeneity test showed no statistical significance in all subgroups (all *P*> 0.05).

**Table 4:** Stratified analysis of the association of the combined effects of *HSPA5*-rs12009, *HSPA5*-rs430397, and *ITPR2*-rs11048570 with NAFLD risk.

Variables	NAFLD group (0/1-3/4-6)	Control group (0/1-3/4-6)	Control group/NAFLD group		P^**^
			OR (95% CI)	P^*^	
Gender					0.221
male	(196/376/156)	(315/594/313)	**0.786 (0.649-0.952)**	**0.014**	
female	(24/39/20)	(190/321/148)	1.129 (0.718-1.775)	0.599	
Age					0.353
≤ 42 years	(117/205/77)	(242/496/225)	0.908 (0.704-1.171)	0.458	
> 42 years	(103/210/99)	(263/419/236)	**0.765 (0.597-0.980)**	**0.034**	
TC					0.318
< 5.72 mmol/L	(175/357/145)	(440/805/406)	0.854 (0.705-1.034)	0.106	
≥ 5.72 mmol/L	(45/57/29)	(64/107/54)	0.671 (0.424-1.062)	0.089	
GLU					0.933
< 5.60 mmol/L	(135/238/93)	(348/659/308)	0.834 (0.666-1.043)	0.111	
≥ 5.60 mmol/L	(85/176/81)	(156/253/152)	0.821 (0.617-1.092)	0.175	
BMI					0.819
< 24 kg/m^2^	(34/175/13)	(213/869/131)	0.818 (0.613-1.092)	0.174	
≥ 24 kg/m^2^	(83/440/38)	(81/460/51)	0.856 (0.664-1.103)	0.230	
TG					0.716
< 1.70 mmol/L	(119/214/97)	(407/729/357)	0.829 (0.661-1.039)	0.104	
≥ 1.70 mmol/L	(101/200/77)	(97/183/103)	0.773 (0.573-1.042)	0.091	

NAFLD, non-alcoholic fatty liver disease; TC, total cholesterol; GLU, glucose; TG, triglyceride; BMI, body mass index.

The stratified factor in each stratum was excluded. The variable age is classified according to the mean and median age of the sample population. The variables TC, GLU, and TG in this table were classified as normal versus abnormal data according to the relevant guidelines. The classification of BMI was based on the level of the Chinese population, and those over 24 kg/m^2^ were considered overweight. Bold type indicates statistically significant results.

^*^ P-values adjusted for sex, age, TC, and GLU by using logistic regression in additive models.

^**^ P-value for the heterogeneity test.

### 3.4 Haplotype analysis of the combined effect of *HSPA5* SNPs on susceptibility of NAFLD

**Figure 1:**
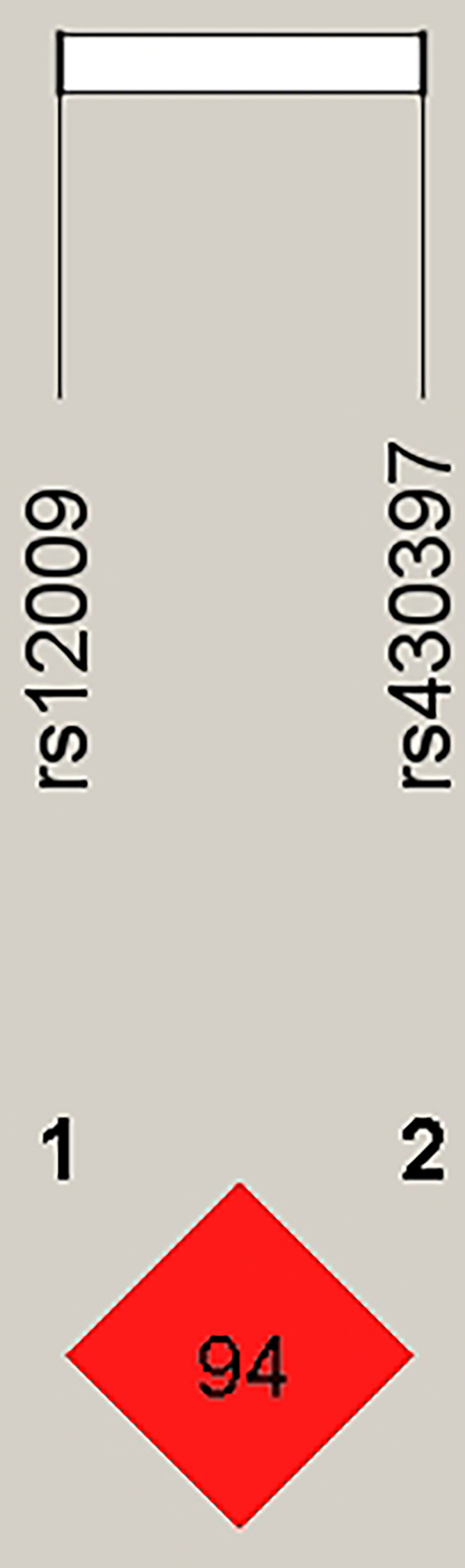
Linkage disequilibrium (LD) plot of *HSPA5*-rs12009 and *HSPA5*-rs430397. The number 98 in the red box represents a strong linkage disequilibrium between *HSPA5*-rs12009 and *HSPA5*-rs430397.

Since the above analyses confirmed that *HSPA5* rs12009 and rs430397 were related to risk of NAFLD, and the genetic loci were all consistent with Hardy Weinberg equilibrium, we further performed haplotype analysis of these SNPs to predict susceptibility to NAFLD. The Linkage disequilibrium (LD) information of these SNPs was shown in **Figure 1** (*D*’= 0.94, *r* ^2 ^= 0.223). Haplotype analysis demonstrated that (**Supplementary Table 4**), four haplotype frequencies were calculated: GC> AC> AT> GT, with the highest frequency of GC in both NAFLD and control populations, but no statistically significant difference in haplotype frequency (*P*> 0.05).

### 3.5 Influence factors of NAFLD

**Figure 2:**
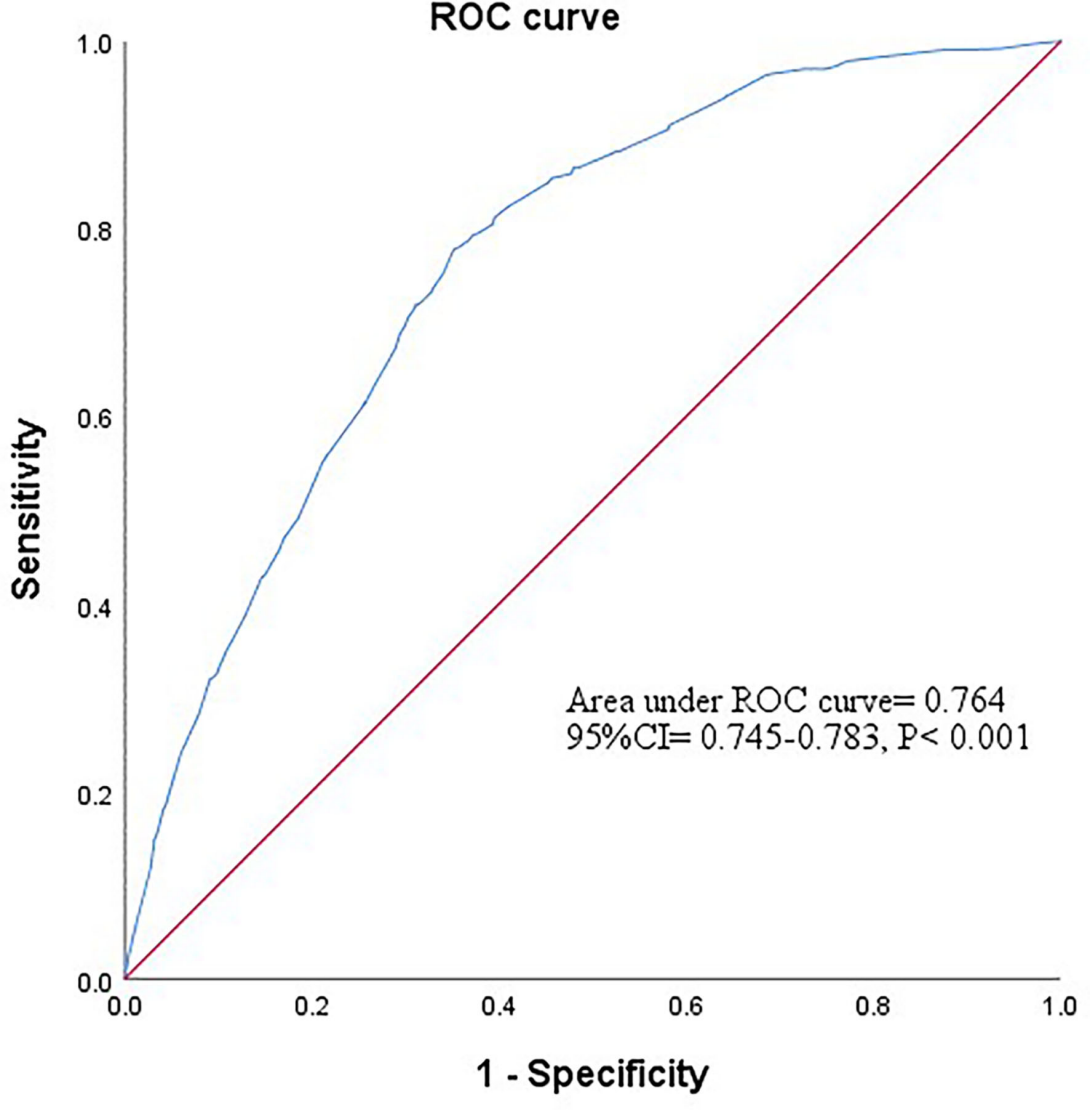
The ROC curve of combined factor model based on the data of gender, glucose, body mass index, triglycerides and favorable alleles. Based on the results of stepwise regression analysis, a combined factor model was constructed for the five variables of gender, glucose, body mass index, triglycerides and favorable alleles to assess the risk of NAFLD. The AUROC for this combined model was 0.764 (95% CI = 0.745-0.783, *P*<0.001).

A stepwise regression model was performed with gender, age, GLU, BMI, TG, TC, and combined favorable alleles (*HSPA5* rs12009-A, *HSPA5* rs430397-T, and *ITPR2* rs11048570-A). The results showed that gender (OR= 0.364, 95% CI= 0.278-0.475, *P*< 0.001) and favorable alleles (OR= 0.926, 95% CI= 0.858-0.999, *P*= 0.047) were independent protective factors of NAFLD. Conversely, higher GLU (OR= 1.368, 95% CI= 1.125- 1.664, *P*= 0.002) and BMI (OR= 3.558, 95% CI= 2.929-4.322, *P*< 0.001), TG (OR= 2.284, 95% CI= 1.879-2.777, *P*< 0.001) were independent risk factors of NAFLD (**Table 5**). Subsequently, based on the 5 variables mentioned above, we constructed a combined factor model to assess the risk of NAFLD. The AUROC of this combined model was 0.764 (95% CI= 0.745-0.783, *P*< 0.001) showed in **Figure 2**. At a cutoff value of 0.427, the sensitivity and specificity of this novel model were 77.8% and 64.9%, respectively. The positive likelihood ratio and negative likelihood ratio were 2.22 and 0.34, respectively. Finally, the results were found to be statistically significant after the Hosmer-Lemeshow test (*P*= 0.091> 0.05), proving that this model showed good agreement between predicted risk and observed outcomes.

**Table 5:** Multivariate stepwise regression analysis for independent factors of NAFLD.

Variables	*b*	*s_ $\bar{\text{x}}$ _*	Wald *χ* ^2^	OR (95% CI)	*P*
Gender (male *vs.* female)	-1.012	0.137	54.789	**0.364 (0.278-0.475)**	< 0.001
Age (≤ 42^†^ *vs.* > 42 years)	-0.005	0.006	0.626	0.996 (0.984-1.007)	0.429
GLU (≤ 5.60^†^ *vs.* > 5.60 mmol/L)	0.314	0.100	9.867	**1.368 (1.125-1.664)**	**0.002**
BMI (< 24^†^ *vs.* ≥ 24 kg/m^2^)	1.269	0.099	163.637	**3.558 (2.929-4.322)**	< 0.001
TG (< 1.70^†^ *vs.* ≥ 1.70 mmol/L)	0.826	0.100	68.765	**2.284 (1.879-2.777)**	< 0.001
Favorable alleles^‡^	-0.077	0.039	3.929	**0.926 (0.858-0.999)**	**0.047**
Constant	-2.750	0.374	54.081		

GLU, glucose; BMI, body mass index; TG, triglycerides; OR, odds ratio; CI, confidence interval; vs., versus.

^†^ The former was used as the reference group in the analysis.

^‡^ Favorable alleles indicate the combination of HSPA5 rs12009-A, HSPA5 rs430397-T, and ITPR2 rs11048570-A. Bold type indicates statistically significant results.

## 4 Discussion

To the best of our knowledge, this was the first study to investigate the association between Ca^2+^ transport protein genetic polymorphisms and the risk of NAFLD. In this case-control study, we found that three SNPs, *HSPA5*-rs12009, *HSPA5*-rs430397 and *ITPR2*-rs11048570, were independent influencing factors for NAFLD. Additionally, rs12009-A, rs430397-T and rs11048570-A were associated with decreased risk of NAFLD as favorable alleles. The discovery and exploration of favorable alleles may provide a new direction for the development of drugs that complement the future treatment of NAFLD.

NAFLD is emerging rapidly as a major global health problem without an approved treatment. There is therefore an urgent demand to identify potential predictors and pharmacological targets. It has been demonstrated that ERS, mitochondrial dysfunction and abnormal lipid metabolism were involved in the pathogenesis of NAFLD (29). Studies have shown that Ca^2+^ homeostasis is related to ERS and mitochondrial dysfunction (30). Moreover, some studies have suggested that dysregulation of Ca^2+^ homeostasis in the MAM region played an important role in hepatic metabolism (31). GRP78 and IP3R2, the proteins encoded by *HSPA5* and *ITPR2* involved in this study, exert important physiological and pathological functions in the MAM region. Regarding GRP78, a master ER chaperone, is also present at the MAM, which is responsible for maintaining the ER permeability barrier during protein translocation, guiding protein folding and assembly, and targeting misfolded proteins for degradation, releasing and activating the unfolded protein response (UPR) sensors to maintain ER homeostasis (32, 33). Several studies have demonstrated that loss of GRP78 in metabolic organs, including the liver, may lead to primary or compensatory outcomes (34–36), such as compensatory ERS, and imbalances in glucose metabolism. Some experiments in GRP78-deficient mice suggested that deficiency of GRP78 may result in susceptibility to various acute or chronic liver diseases, and that GRP78 may be a regulator of hepatic steatosis and hepatocellular carcinoma. In MAM, IP3R2 resides near the ion channel VDAC, located in the outer mitochondrial membrane. This structure facilitates Ca^2+^ uptake into the mitochondrial matrix *via* the low-affinity mitochondrial Ca^2+^ uniporter (MCU) (37). Furthermore, IP3R2 is the major Ca^2+^ release channel of MAM in hepatocytes (38), may play an important role in lipid homeostasis, as it has been found that *ITPR* mutant Drosophila store excess triglycerides and become obese even on a normal diet (39). Furthermore, one study showed that *ITPR2* expression was significantly reduced or absent in patients with steatosis and NASH (40). However, several works indicated that IP3R2 was a driver of senescence in human cells (20, 41–44), and *ITPR2* knockout mice had suppressed Ca^2+^ fluxes in the MAM region and displayed less immunosenescence as well as less hepatic steatosis and fibrosis (20). Besides, some studies of human HCCs discovered that hepatitis B virus (HBV) DNA targets integrated hepatocyte DNA, while *ITPR2* showed a high prevalence of HBV integration, which may indicate that *ITPR2* plays an important role in hepatocarcinogenesis (45, 46).

Although there is no evidence directly suggesting that genetic polymorphisms in the Ca^2+^ transport protein of this study are associated with NAFLD outcomes, these genetic variants continue to play an essential role in ERS and mitochondrial dysfunction, and have been extensively explored in other diseases (47–49). It was found that *HSPA5*-rs12009 was significantly associated with knee osteoarthritis (KOA) severe progression in an exploratory cohort (50). Further presented studies suggested that *HSPA5*-rs430397 was associated with ERS (51). Meanwhile, a case-control study showed that *HSPA5*-rs430397 effectively predicted the primary hepatocellular carcinoma (52) and liver fibrosis (53), which also complemented our study hypothesis and results. Moreover, there are experimental results showing that the locus *ITPR2*-rs11048570 is significantly associated with Kashin-Beck Disease in the Han Chinese. It seems to be no direct connection between *ITPR2*-rs11048570 and NAFLD in previous studies, but they have confirmed the importance of *ITPR2* in regulating apoptosis. It is observed that apoptotic stimuli could trigger IP3R mediated Ca^2+^ signaling, leading to mitochondrial release of cytochrome C and initiation of the complete apoptosis program (54). It is also demonstrated that phosphorylation of IP3Rs diminished Ca^2+^ flux from ER to mitochondria and significantly reduced cellular sensitivity to apoptotic stimuli (55). Although the potential molecular mechanism of *ITPR2* involved in the development of NAFLD remains unclear, it is reasonable to speculate that *ITPR2* contributes to abnormal hepatocyte apoptosis in the liver of patients with NAFLD. This article complements the association studies of *HSPA5*-rs12009, *HSPA5*-rs430397 and *ITPR2*-rs11048570 with NAFLD to some extent.

In addition, we found that combined effects of rs12009-A, rs430397-T, and rs11048570-A alleles were associated with the decreased risk of NAFLD. And as a result of stratified analysis, our report showed that the combined effects of these gene variants were statistically significant in the male and the age> 42 years subgroup, and there was no significant heterogeneity within each subgroup variable, suggesting that these variables did not alter this association. In fact, some studies have pointed to a higher incidence of NAFLD in men, and the gender differences may be related to differences in hormone, serum TG, and blood glucose levels (56). Besides, the prevalence of NAFLD increases with age and studies have speculated that the increased risk of NAFLD may contribute to the high prevalence of metabolic diseases in the elderly (57). These above studies are all consistent with our findings.

This study also demonstrated that favorable alleles were independent protective factors of NAFLD; male, higher GLU, BMI and TG levels were independent risk factors of NAFLD, which is accordance with the results of previous studies (56, 58–60). We combined these independent factors to assess NAFLD risk and the AUROC for this combined model was 0.764 (*P*< 0.001). The results showed that combined factors described above were efficient in assessing NAFLD risk, which may provide new approaches for early screening of high-risk populations.

Although this study was the first to explore the association of *HSPA5* and *ITPR2* gene polymorphisms with susceptibility to NAFLD and obtained some meaningful results, there were still several limitations. First, the representation of our sample may be insufficient. The subjects enrolled in this study were from a single community with a relatively small female population. To address this issue, the frequency-matching of gender and age was used in the design stage, and multivariate analysis and stratified analysis were utilized to minimize the effects of confounding factors. Second, we selected only a few key genes in the numerous pathways of Ca^2+^ transport, which may not adequately analyze the relationship between genetic factors and the risk of NAFLD. Thus, we attempted to use haplotype analysis, which laid the foundation for completing genetic information of related SNPs and searching for haplotypes associated with NAFLD, which was important for the study of Ca^2+^ transporter gene polymorphisms and their combinatorial distribution and the role of haplotype genotype distribution in disease mechanisms. Third, in view of the complexity of the pathogenesis of NAFLD, it is necessary to further explore the impact of gene polymorphisms and their combination with environmental factors on disease risk in a multicenter population of different races. Finally, the biological mechanism by which SNPs are related to the outcome of NAFLD is poorly understood. Therefore, replication studies with functional characterization of these SNPs in various lager multicenter population of different races are required to confirm our findings.

## 5 Conclusion

In conclusion, our findings suggested that gene polymorphisms of Ca^2+^ transport proteins and molecular chaperones play an important role in the development of NAFLD, particularly when associated with older age or males. Overall, *HSPA5* gene (rs12009, rs430397) and *ITPR2* gene (rs11048570) were associated with susceptibility to NAFLD within Chinese population.

## Data availability statement

The original contributions presented in the study are included in the article/**Supplementary Material.** Further inquiries can be directed to the corresponding author.

## Ethics statement

The studies involving human participants were reviewed and approved by Institutional Ethics Review Committee of Nanjing Medical University. The patients/participants provided their written informed consent to participate in this study.

## Author contributions

ZT and YD: Data curation, Formal analysis, Investigation, Writing – original draft, Writing – review & editing. RZ, MZ, QG and LZ: Data curation, Investigation, Software. HW, YC, RJ and WZ: Investigation, Methodology, Resources. JW: Conceptualization, Funding acquisition, Project administration, Supervision, Writing – review and editing. All authors contributed to the article and approved the submitted version.
