# Supplementary material for: Genetic polymorphisms of Ca2+ transport proteins and molecular chaperones in mitochondria-associated endoplasmic reticulum membrane and non-alcoholic fatty liver disease
Source: Front Endocrinol (Lausanne). 2023 Jan 4;13:1056283. doi: 10.3389/fendo.2022.1056283 (PMC9846251; doi:10.3389/fendo.2022.1056283)
Supplement: Supplementary file 1 [file DataSheet_1.docx]

Supplementary Material

# Supplementary Tables

## Supplementary Table 1

| **Supplementary Table 1**. Information of key Ca^2+^ transport genes and encoded proteins in the MAM region | | | | | | | | |
| --- | --- | --- | --- | --- | --- | --- | --- | --- |
| Variables | Gene | Encoded protein | Chromosome | Starting site | Termination site | protein expression^†^ | RNA expression  (nTPM)^‡^ | Whether expressed in liver tissue |
| Transporter | *ITPR2* | IP3R2 | chr12 | 26335337 | 26833198 | Medium | 13.3 | Yes |
|  | *VDAC1* | VDAC1 | chr5 | 133971875 | 134070987 | Medium | 206.2 | Yes |
| Chaperone | *HSPA9* | GRP75 | chr5 | 138554882 | 138575629 | High | 194.2 | Yes |
|  | *HSPA5* | GRP78 | chr9 | 125234848 | 125241387 | Medium | 327.8 | Yes |
|  | *SIGMAR1* | SIG1R | chr9 | 34634722 | 34637826 | High | 187.6 | Yes |
|  | *CANX* | Calnexin | chr5 | 179678646 | 179731641 | Medium | 193.2 | Yes |
|  | *PPID* | CYPD | chr4 | 158709127 | 158723400 | Medium | 87.6 | Yes |
| *Abbreviations*: Ca^2+^, calcium; MAM, mitochondria-associated endoplasmic reticulum.  ^†^ Protein expression scores are divided into four levels: High, Medium, Low and Not.  ^‡^ RNA expression is measured in nTPM (normalized protein-coding transcripts per million). | | | | | | | | |

## Supplementary Table 2

| **Supplementary Table 2**: SNPs and functional prediction of key Ca^2+^ transport genes in the MAM region. | | | | | |
| --- | --- | --- | --- | --- | --- |
| Gene | SNPs | Site | Alleles | MAF | Regulome DB^†^ score |
| *HSPA5* | rs1140763 | 3 Prime UTR Variant | G> A | 0.443 | 3a |
|  | rs12009 | 3 Prime UTR Variant | G> A | 0.100 | 1b |
|  | rs430397 | Intron Variant | C> T | 0.122 | 3a |
| *ITPR2* | rs10771283 | Intron Variant | G> A | 0.387 | 3a |
|  | rs11048570 | Intron Variant | G> A | 0.178 | 4 |
|  | rs2230372 | Intron Variant | G> A | 0.415 | 4 |
| *Abbreviations*: SNPs, single nucleotide polymorphisms; Ca^2+^, calcium; MAM, mitochondria-associated endoplasmic reticulum; MAF, Minor allele frequency.  ^†^ Regulome DB is a database for screening potential functional loci. | | | | | |

## Supplementary Table 3

| **Supplementary Table 3**: Association of 6 SNPs with NAFLD risk in dominant, recessive, and addictive model of multivariable analysis. | | | | | | | | | | |
| --- | --- | --- | --- | --- | --- | --- | --- | --- | --- | --- |
| SNPs | Co-dominant model  (GA vs. GG) | | Co-dominant model  (AA vs. GG) | | Dominant model  (GA+ AA vs. GG) | | Recessive model  (AA vs. GA+ GG) | | Addictive model  (AA vs. GA vs. GG) | |
|  | *P* | *P*_FDR_^*^ | *P* | *P*_FDR_^*^ | *P* | *P*_FDR_^*^ | *P* | *P*_FDR_^*^ | *P* | *P*_FDR_^*^ |
| rs1140763 (G> A) | 0.406 | 0.940 | 0.202 | 0.303 | 0.269 | 0.571 | 0.314 | 0.471 | 0.193 | 0.290 |
| rs12009 (G> A) | 0.940 | 0.940 | 0.086 | 0.172 | 0.525 | 0.571 | **0.036** | **0.098** | 0.098 | 0.290 |
| rs430397 (C> T) | 0.784 | 0.940 | **0.031** | **0.138** | 0.423 | 0.571 | **0.032** | **0.098** | 0.163 | 0.290 |
| rs10771283 (G> A) | 0.470 | 0.940 | 0.978 | 0.978 | 0.571 | 0.571 | 0.703 | 0.703 | 0.851 | 0.290 |
| rs11048570 (G> A) | 0.733 | 0.940 | **0.046** | **0.138** | 0.373 | 0.571 | **0.049** | **0.098** | 0.134 | 0.290 |
| rs2230372 (G> A) | 0.287 | 0.940 | 0.967 | 0.978 | 0.406 | 0.571 | 0.634 | 0.703 | 0.736 | 0.290 |
| *Abbreviations*: SNPs, single nucleotide polymorphisms; FDR, false discovery rate.  Bold type indicates statistically significant results, deriving from logistic regression analyses with adjustment for gender, age, TC, and GLU.  ^*^ Adjusted *P*-value using FDR for multiple testing correction. The *P*_FDR_ value≤ 0.25 was regarded as modest confidence that the correlation represented a positive result. | | | | | | | | | |  |

## Supplementary Table 4

| **Supplementary Table 4**: Haplotype distributions of *HASP5*-rs12009 and rs430397 in NAFLD group and Control group. | | | | |
| --- | --- | --- | --- | --- |
| Haplotype | NAFLD group  No. (%) | Control group  No. (%) | OR (95%CI) | *P^*^* |
| GC | 850 (52.1) | 1917 (50.8) | 1.000 (ref.) |  |
| GT | 9 (0.6) | 13 (0.3) | 1.707 (0.699-4.168) | 0.240 |
| AC | 483 (29.6) | 1124 (29.8) | 0.933 (0.811-1.074) | 0.334 |
| AT | 288 (17.7) | 718 (19.0) | 0.864 (0.732-1.021) | 0.085 |
| *Abbreviations*: NAFLD, non-alcoholic fatty liver disease; OR, odds ratio; CI, confidence interval.  Logistic regression analysis was adjusted for age, gender, TC, and GLU. Bold type indicates statistically significant results.  ^*^ NAFLD individuals *versus* controls. | | | | |
